# Cu^2+^ Release from Polylactic Acid Coating on Titanium Reduces Bone Implant-Related Infection

**DOI:** 10.3390/jfb13020078

**Published:** 2022-06-10

**Authors:** Chengdong Zhang, Xingping Li, Dongqin Xiao, Qiao Zhao, Shuo Chen, Fei Yang, Jinhui Liu, Ke Duan

**Affiliations:** 1 Research Institute of Tissue Engineering and Stem Cells, Nanchong Central Hospital, The Second Clinical College of North Sichuan Medical College, Nanchong 637000, China; xd_zcd1@163.com (C.Z.); joe_zhow@163.com (Q.Z.); csdoc1212@163.com (S.C.); yang1190fei@163.com (F.Y.); 2 Key Laboratory of Advanced Technologies of Materials (MOE), School of Materials Science and Engineering, Southwest Jiaotong University, Chengdu 610031, China; 3 Department of Orthopaedics, Chengfei Hospital, Chengdu 610091, China; 13488956158@163.com; 4 Sichuan Provincial Laboratory of Orthopaedic Engineering, Department of Bone and Joint Surgery, Affiliated Hospital of Southwest Medical University, Luzhou 646000, China; jinhui_liu19830514@163.com

**Keywords:** bone, implant, infection, coating, copper

## Abstract

Implant-related infection (IRI) is a major problem in orthopedics. Copper (Cu) is an essential trace element with strong bactericidal activity and, thus, presents potential for reducing IRI. The present study explored a straightforward strategy for releasing Cu^2+^ from titanium (Ti) implants, and we conducted a preliminary study to assess the feasibility of this approach in clinical translation. Polylactic acid (PLA) coatings containing different concentrations of copper ions were prepared on Ti discs. The antibacterial activity and biocompatibility of the copper ion-incorporated Ti implants were evaluated using Staphylococcus aureus (*S. aureus*), bone marrow mesenchymal stem cells (BMSCs) and animal models. In vitro, the coatings produced burst release of Cu^2+^ in 12 h, and inhibited *S. aureus* growth in a dose-dependent manner. The coatings prepared from PLA solutions containing 0.5 or 1.0 mg/mL reduced the viability and osteogenic differentiation of BMSCs, but these effects were negated after the coatings were immersed in culture medium for 6 h. Four weeks after implantation, the Cu-free K-wires challenged with *S. aureus* had persistent infection and inferior fracture healing to the other three groups, while Cu-coated wires had no evidence of infection. Furthermore, the Cu-coated wires placed in rabbits without *S. aureus* challenge showed superior fracture healing to the other three groups. These results suggest that PLA coatings containing Cu^2+^ may be an effective design for reducing IRI without adversely affecting adjacent bone healing.

## 1. Introduction

Implant-related postoperative infection (IPI) is a significant risk associated with orthopedic surgeries, with estimated incidences of 0.3–3% [1]. The leading cause of IPIs is Staphylococcus *aureus* (*S. aureus*), followed by *E. coli*, *S. epidermidis*, and other pathogens (e.g., fungi) [2,3]. Although the incidence of IPI seems relatively low, its treatment is difficult, lengthy, and costly. To prevent IPI, many techniques have been developed to endow implant surfaces with antibacterial activity. Vester et al. reported a gentamicin-loaded polylactic acid (PLA) coating on titanium (Ti) pins [4]. Coated intramedullary tibial nails have entered clinical application in Europe and were reported to effectively reduce postoperative infections associated with high-risk fractures and complex nonunion cases [5]. However, globally, the prophylactic use of antibiotics is increasingly restricted in clinics because of concerns over the development of antibiotic resistance. Additionally, an antibiotic is effective towards only a spectrum of microorganisms. For example, gentamicin is most effective for Gram-negative bacteria (e.g., *E. coli*), and is partly effective for Gram-positive ones (e.g., *S. aureus*) and ineffective for fungi.

Inorganic antibacterial substances represent another possibility to control IPI [6]. As opposed to antibiotics, inorganic substances are not Gram-specific and are unable to induce antibiotic resistance. A potent bactericidal element, silver (Ag), has been extensively studied for reducing IPI [7,8,9]. However, as a moderately toxic and non-essential element for humans, the application of Ag to orthopedic implants remains limited. Copper (Cu) is another potent bactericidal element against both bacteria and fungi [10,11]. As opposed to Ag, Cu is an essential trace element for humans, with an average daily dietary requirement of 2 mg for adults. Owing to its antibacterial activity and relative safety, earlier studies developed several techniques to incorporate Cu into orthopedic implants. Liu et al. prepared a new Ti alloy containing 5% (*w*/*w*) Cu, and demonstrated that the Cu^2+^ release for >35 d could inhibit oral bacteria survival and biofilm formation [11]. However, it was unclear whether Cu^2+^ release from this alloy would proceed indefinitely or terminate after a certain period. From clinical considerations, long-lasting Cu^2+^ release is unnecessary and even undesirable. Furthermore, many other properties of the alloy need to be well characterized before it can be considered for implant production, such as mechanical properties (e.g., fatigue), corrosion rate, and forming ability. Therefore, more studies have been focused on surface modification techniques. Wan et al. implanted Cu^2+^ into stainless steel and Ti by ion-beam implantation [12]; the implanted metals exhibited antibacterial activity towards *S. aureus* but also inferior corrosion resistance relative to the unmodified metals. Moreover, the span of Cu^2+^ release (e.g., duration of release) from these modified metals remains unclear. Norambuena et al. coated TiCuO thin films containing 20–80% Cu (*w*/*w*) on Ti6Al4V by magnetron sputtering, and found that in vitro Cu release peaked at 24 h and was sustained for >28 d [13]. The films reduced the viability of *S. epidermidis* in a Cu-dose-dependent manner; the film containing 80% Cu moderately reduced the survival of human osteoblasts at 24 h, but the cytotoxicity disappeared on day 7. However, release for 28 d is probably unnecessary, and magnetron sputtering requires special high-vacuum facilities and complex technical controls, which may adversely affect the translation of the technique to the batch manufacturing of implants. Wang et al. deposited Cu^2+^ on Ti substrates precoated with polydopamine [14]. The Cu-deposited Ti rapidly released Cu^2+^ in several hours in vitro, and inhibited bacterial infection and promoted osteointegration in a rat metaphyseal implantation model. However, uncertainties and concerns exist for polydopamine, such as its unclear metabolites and metabolic pathways, the formation of side products during polymerization, and unintended organocatalytic actions in vivo [15,16].

Given the successful translation of gentamicin-loaded PLA coatings to clinics [5], the release of Cu^2+^ from a biocompatible polymer coating appears a simple, straightforward approach to reducing IPIs. Because of its simplicity, it may also be technically convenient to control the dose of Cu^2+^ loaded and its release kinetics. Expectedly, such coatings may be applied to osteosynthetic devices (e.g., fixation plates, intramedullary nails), which frequently face the risk of fracture-site infection, and does not require a permanent strong fixation in the host bone. However, this simple design has not been investigated previously. Therefore, we conducted a preliminary study to evaluate the feasibility of this approach. The aims of the present study were to: (1) characterize the relation between Cu^2+^ dose and antibacterial activity and in vitro biocompatibility; (2) test the effect of the coating in a small animal bone fracture model.

## 2. Materials and Methods

### 2.1. Coating Preparation

Commercially pure Ti discs (Φ10 × 1 mm; Xingye Metals, Qinghe, China) were abraded to a 1200-grit finish with silicon carbide sandpaper, and sonicated sequentially in acetone, ethanol, and distilled water (10 min in each). They were etched in a mixture of 0.5 M nitric acid and 0.3 M hydrofluoric acid for 30 s, rinsed and sonicated in ultrapure water (18.2 MΩ.cm), and air-dried. Anhydrous copper chloride (CuCl_2_, analytical grade, Kelong Chemical, Chengdu, China) was manually ground and sieved with a 300-mesh screen. The particles passing the screen were collected.

Poly-D, L-lactide (PDLLA; 0.8 g; Mw: 62 k; Daigang Biotech, Jinan, China) was dissolved in 10 mL of dichloromethane (DCM; Kelong Chemical, Chengdu, China) with the assistance of sonication in an ice bath. Then, 0, 1, 5, or 10 mg of CuCl_2_ was added to the solution, and the tube was sonicated to disperse the particles. A Ti disc was vertically dipped into the suspension, maintained for 15 s, withdrawn (~5 mm·s^−1^), and dried in a fume hood. The process was repeated 2 times. Five groups of samples were prepared: Group A (uncoated Ti discs), Group B (Ti discs coated in PDLLA/DCM solution containing no CuCl_2_), and Groups C-E (Ti discs coated in PDLLA/DCM solution containing 0.1, 0.5, or 1.0 mg/mL CuCl_2_).

### 2.2. Coating Characterizations

Coated discs were sputter-coated with gold and observed by scanning electron microscopy (FEI Quanta 200). For in vitro Cu^2+^ release, samples from Groups C–E were each placed in 2 mL of Dulbecco’s modified Eagle medium (DMEM, Gibco, Gaithersburg, MD, USA) and incubated (37 °C; Thermo Fisher i160) for up to 7 d. At planned intervals, 1 mL of fluid was collected for Cu^2+^ quantitation by inductively coupled plasma mass spectrometry (ICP-MS, NexION 350X, Perkin-Elmer, Waltham, MA, USA) and the original medium was replenished. Contact angle was measured by placing a 5-µL water drop on the sample surface and imaging it for drop shape analysis (ZJ-6900, Zhijia Instrument, Shenzhen, China). Surface roughness was determined with a confocal laser microscope (VK-X1000, KEYENCE, Japan; scan range: 1.4 × 1.1 × 0.2 mm^3^) and expressed as Ra.

### 2.3. In Vitro Antibacterial Tests

Discs were also tested for the inhibition of *S. aureus* (BNCC186335; BeNa Culture Collection company). Briefly, 0.5 mL of liquid bacterial suspension (BNBio, Beijing, China) was pipetted into 5 mL of presterilized (121 °C × 15 min) liquid culture medium (10.0 g peptone, 5.0 g beef extract (both SolarBio, Beijing, China), 5.0 g sodium chloride, 1000 mL water) and thoroughly mixed. The resulting liquid was streaked on agar medium (100 mL of liquid medium and 14.0 g agar). After incubation (37 °C) for 24 h, one colony was picked, transferred into 4 mL of sterilized liquid medium, and maintained in a shaking incubator (37 °C, 150 r/min) for 24 h. The bacterial suspension was diluted to 10^5^–10^7^ CFU/mL for subsequent use. Then, Ti discs were sterilized by UV irradiation for 12 h and placed in 6-well dishes; 100 μL of the bacterial suspension was pipetted on each disc. After incubation of the dish (37 °C) for 20 min, warm (45 °C) agar medium was poured into each well, gently swirled to promote mixing, and allowed to cool to room temperature and solidify. After incubation (37 °C) for 12 h, the dish was visually examined for bacterial growth.

For observing the bacterial morphology on the Ti discs, bacterial suspension (100 μL) was pipetted on discs and incubated (37 °C) for 6 h. They were fixed with 4 mL of 2.5% glutaraldehyde (4 °C, 6 h), dehydrated in ethanol solutions (60%, 70%, 80%, 90%, 100%, 30 min in each), and dried (37 °C, 12 h). Finally, they were studied by scanning electron microscopy (SEM).

### 2.4. Quantitative Bacterial Inhibition

UV-sterilized Ti discs were placed in 6-well dishes; 100 μL of the bacterial suspension was pipetted on each disc, and dishes were incubated (37 °C) for 20 min. Then, 4 mL of the liquid culture medium was added to each well, and the dish was placed in a shaking incubator (37 °C, 150 r/min). After 0, 0.5, 2, and 6 h, the suspension was collected and diluted up to 200-fold with sterile normal saline; 100 μL of the dilution was spread on agar. After incubation (37 °C, 12 h), the colonies formed were counted and converted to the original bacterial concentration (in CFU·ml^−1^). For a Group X (X = A, B, C, D, or E), the bacteria survival rate was calculated by: (CFU·ml^−1^ for Group X/CFU·ml^−1^ for Group B) × 100.

### 2.5. Cytocompatibility

Biocompatibility of the coated samples was studied under two scenarios: (1) direct seeding of bone marrow mesenchymal stem cells (BMSCs) on the samples (Scenario 1) and (2) pre-elution of each sample in 4 mL of DMEM for 6 h before seeding of BMSCs (Scenario 2). As previous experiments found that all coatings released their CuCl_2_ loads in ~6 h (see Results), examination under the two scenarios allowed us to better understand the biocompatibility of the coatings both during and after their Cu^2+^ release. Briefly, rabbit BMSCs at third passage were purchased from the Cell Bank of Institute of Zoology, Chinese Academy of Sciences (Kunming, China). BMSCs (1 × 10^5^ cells/well) were seeded onto UV-sterilized Ti discs cultured in DMEM containing 10% fetal bovine serum. After incubation for 1 d, 3 d or 5 d, fluid in 3 wells of each group was discarded and replaced with 200 μL of DMEM supplemented with 10% (*v*/*v*) CCK-8 cell-counting reagent (KeyGen Biotech, Nanjing, China). After incubation for 2 h, the absorbance at 450 nm was measured on Thermo Fisher Varioskan Flash (Thermo Fisher Scientific, Waltham, USA).

In addition, expression levels of four osteogenic genes (osteopontin (OPN), alkaline phosphatase (ALP), osteocalcin (OCN), and osterix (OSX)) were measured by quantitative PCR. UV-sterilized Ti discs, with or without undergoing a 6 h pre-immersion in 4 mL of DMEM, were placed in six-well culture plates (4 discs/group, 3 wells/group); 1 × 10^6^ BMSCs were added to each well, and the plates were incubated for 14 d with daily renewal of the medium. Subsequently, RNA was isolated using Trizol (Invitrogen) and reverse-transcribed to cDNA using first-strand cDNA kits (Invitrogen) following the manufacturer’s instructions. Then, the cDNAs were amplified by qPCR with SYBR Green (Bio-Rad Laboratories, Inc.) on an iQ5 multicolor real-time PCR detection system (Bio-Rad Laboratories) using corresponding primers (Table 1). The PCR conditions were as follows: 95 °C for 3 min followed by 40 cycles of 95 °C for 15 s and 60 °C for 15 s. All experiments were performed in triplicate and the relative expression levels of genes were quantified by the 2-ΔΔCq method [17], using the glyceraldehyde-3-phosphate-dehydrogenase (GAPDH) gene as the control.

### 2.6. In Vivo Model

In vitro experiments observed that (see Results) Group E offered the highest antibacterial activity and acceptable cytocompatibility after pre-elution for 6 h. Thus, an in vivo model was used to evaluate its anti-infective function. All procedures were approved by the Animal Ethics Committee of North Sichuan Medical College (NSMC(A)2020(10)). Twenty-four male New Zealand rabbits (weight: ~2.5 kg, age: 3 months; Center of Laboratory Animals, North Sichuan Medical College) were randomized to four groups (n = 6/group) (Table 2). Ti6Al4V Kirschner wires (Φ2 × 90 mm) were coated following Section 2.1 and sterilized by UV irradiation for 24 h. The animal was anesthetized by intravenous injection of 2% pentobarbital sodium (1.5 mL/kg). A Kirschner wire (Φ1.5 mm) was inserted from 0.5 cm below the right tibial plateau and advanced to the distal end. The medullary canal was enlarged with another K-wire (Φ2.5 mm). The tibia was transected at the middle, and 50 μL of saline or *S. aureus* suspension (Table 2) was carefully injected into the proximal and distal stumps, respectively (total: 100 μL). The Ti6Al4V wire was inserted via the same entry point as the K-wire to fix the tibia. Then, the wound was sutured layer by layer. After operation, the animal received intramuscular penicillin for 3 d (10,000 IU/kg, one injection per day), and the operated limb was immobilized for 4 weeks by casting.

Moreover, the animal was regularly checked for food/water intake and physical activity. Rectal temperature was monitored regularly. Blood sample was collected weekly and assayed for white blood cell count (WBC), C-reactive protein (CRP), procalcitonin (PCT), and copper. Four weeks after operation, the animal was killed by intravenous pentobarbital overdose. After radiographic examinations, the operated tibia was dissected and stripped of soft tissues. The wires were removed from the proximal end of the tibia under aseptic conditions and smeared with a wet cotton ball. The cotton ball was gently rolled over agar medium, incubated (37 °C) for 24 h, and examined for colony formation. Fracture healing was evaluated by radiography and callus index (i.e., maximum diameter of callus divided by the bone diameter [18]). For histological examination of bone healing, samples were fixed in formalin (10%, 1 week) and decalcified in ethylenediamine tetratacetic acid sodium (10%, 37 °C) for up to 2 months until a needle could easily penetrate the sample. It was dehydrated in a series of ethanol solutions (60%, 70%, 80%, 90%, 100%, 30 min for each), embedded in paraffin, cut longitudinally into slices (3 µm), and stained with hematoxylin–eosin. Fracture healing was scored using a five-degree scoring system [19] (scores 0: pseudojoint formation and cavity between fracture ends; 1: incomplete cartilaginous healing with fibrosis; 2: complete cartilaginous healing between fracture fragments and the formation of hyaline cartilage plates; 3: incomplete bony union with residual cartilage in callus; 4: complete bony union, with fracture ends connected by mature trabeculae).

### 2.7. Statistical Analysis

Data were analyzed by analysis of variance (ANOVA) followed by Tukey test (SPSS 18.0, SPSS, Chicago, IL, USA). A *p*-value < 0.05 was considered statistically significant.

## 3. Results

### 3.1. Morphology and In Vitro Cu^2+^ Release

**Figure 1:**
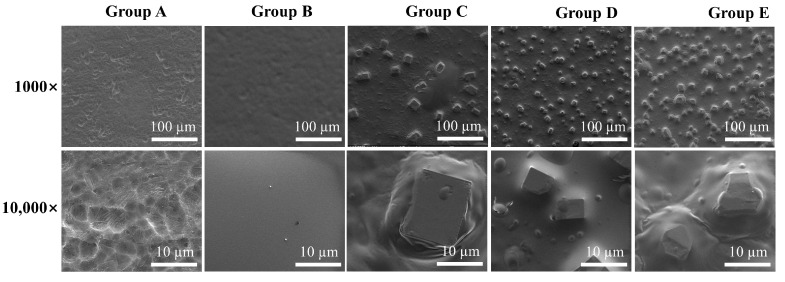
SEM images of the different samples (Group A: uncoated Ti discs, Group B: Ti discs coated in PDLLA/DCM solution containing no CuCl_2_, and Groups C–E: Ti discs coated in PDLLA/DCM solution containing 0.1, 0.5, or 1.0 mg/mL CuCl_2_).

Under SEM (Figure 1), Group A showed a generally flat surface with micrometer-wide pits produced by the etching treatment. Group B had a smooth surface lacking surface features, as a result of coverage by a PDLLA coating. Groups C–E exhibited cubic particles (size: 5~10 μm) of increasing density embedded in a featureless matrix. Severe CuCl_2_ particle agglomeration was not observed in any group. The contact angle of uncoated Ti (Group A) was 67.5 ± 5.2° (n = 3). The contact angles of Groups B–E increased to 71.9 ± 1.6°, 78.9 ± 1.8°, 72.4 ± 0.8°, and 73.9 ± 8.0° (all n = 3) (Supplementary Figure S1a). The slight increase in contact angle coincided with the more hydrophobic PDLLA coating. Ra values for Groups A–E were 0.74 ± 0.02, 0.69 ± 0.05, 0.83 ± 0.08, 1.07 ± 0.21, and 2.07 ± 0.21 µm, respectively (Supplementary Figure S1b). This trend was consistent with the results that the smooth PDLLA coating decreased the Ti surface roughness, while CuCl_2_ particle addition increased the surface roughness.

**Figure 2:**
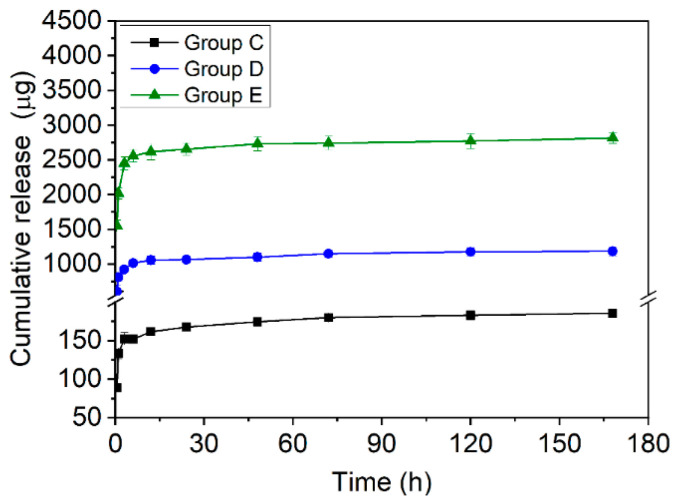
Cumulative Cu ion release of the different samples.

During immersion in DMEM, Groups C–E (Figure 2) produced a rapid initial release in the first 12 h, followed by a slower release tending to a plateau. The cumulative release of Cu^2+^ showed a dose-dependent trend of Group E > Group D > Group C. Generally, the cumulative release of Group C reached 162 ± 4 µg at the first 12 h, while Groups D and E reached 1055 ± 60 µg and 2615 ± 115 µg at ~12 h. After 7 d, the cumulative release for Group C was 186 ± 4 µg, while the results for Groups D and E were 1183 ± 66 µg and 2814 ± 78 µg. Throughout the experiment, no direct liberation of CuCl_2_ particles from the coating was visually observed, confirming that Cu^2+^ was released via dissolution of these particles, as may be expected from the high solubility of CuCl_2_ (~77 g/L). Moreover, all coatings survived immersion for several days without observable detachment or fragment liberation. The SEM study on day 2 (Supplementary Figure S2) revealed that, in Group E, the dissolution of CuCl_2_ particles during in vitro release created circular defects in the coating, but it remained adhered to the substrate.

### 3.2. In Vitro Antibacterial Properties

**Figure 3:**
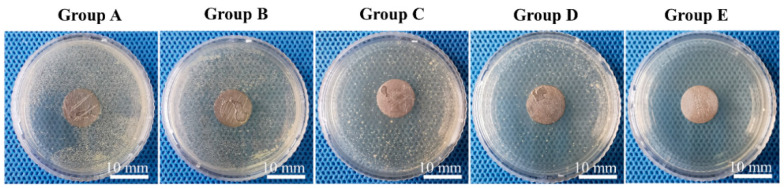
In vitro *S. aureus* inhibition.

After incubation of inoculated Ti discs in agar medium for 12 h (Figure 3), Groups A and B formed large numbers of colonies. Groups C and D formed progressively fewer colonies, with the peri-disc region seeming to have a lower colony density compared with the more distant region. No colony was observed in Group E. SEM showed that, after incubation for 6 h (Supplementary Figure S3), in Group A, a large number of clustered grape-like *S. aureus* cells attached to the surface. In Group B, the cells were partly entrapped in the surface pores of the coating. From Groups C to E, the bacterial density decreased and their morphology changed. In particular, in Group E, the *S. aureus* cells appeared ruffled, in contrast to the smooth and plump morphology seen in Group A.

**Figure 4:**
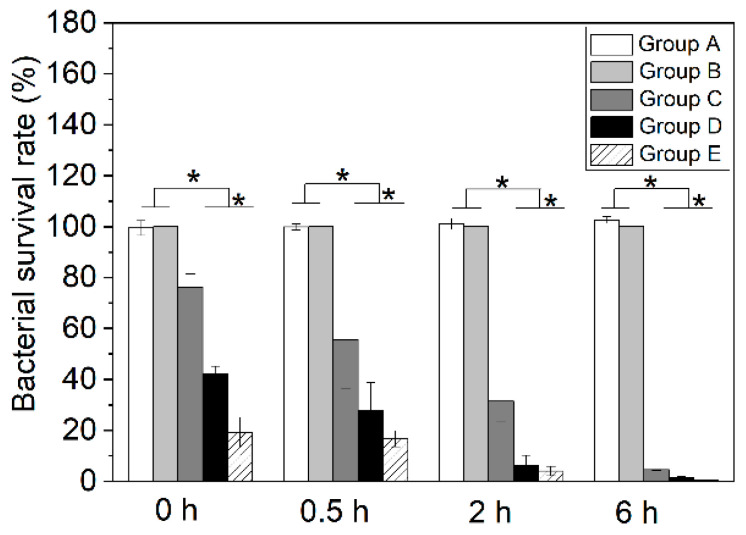
Percentage of viable *S. aureus* after co-culture with different samples for various times (* represents *p* < 0.01).

The quantitative bacterial inhibition test found that (Figure 4), compared with Groups A and B, Groups C–E progressively reduced *S. aureus* survival in both time- and Cu^2+^-dose-dependent manners. After culture on the surfaces of different samples, the bacterial survival rates were reduced from 99.5 ± 2.9% (Group A) to 76.3 ± 5.2% (Group C), 42.3 ± 2.8% (Group D), and 19.2 ± 6.0 (Group E). After shaking for 6 h, the survival rates were further reduced to 4.6 ± 0.4% (Group C), 1.6 ± 0.3% (Group D), and 0.3 ± 0.0% (Group E), compared with 102.6 ± 1.3% in Group A. At any time point studied, the difference between any Cu^2+^-loaded group and each of the other two groups was statistically significant (all *p* < 0.01).

### 3.3. Biocompatibility

**Figure 5:**
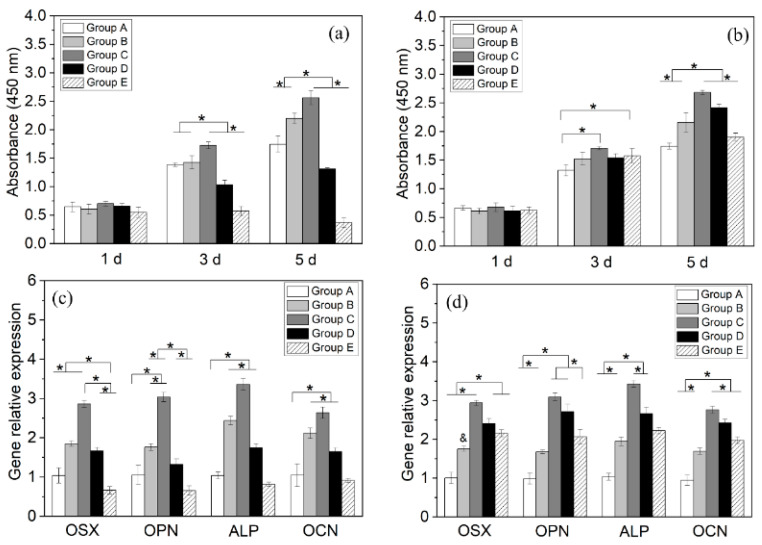
Viabilities of BMSCs seeded on Ti discs (**a**) directly (Scenario 1) or (**b**) after pre-elution in 4 mL of DMEM for 6 h (Scenario 2); expression levels of osteogenic genes of BMSCs seeded on Ti discs (**c**) directly (Scenario 1) or (**d**) after pre-elution in 4 mL of DMEM for 6 h (Scenario 2) (* represents *p* < 0.05).

When directly seeded on the samples (i.e., Scenario 1 in Section 2.5), the BMSCs in Groups A–D proliferated between 1 and 5 d, whereas those in Group E decreased (Figure 5a). Throughout the study, Group C had the highest number of viable cells, and Groups C–E exhibited a CuCl_2_-dose-dependent decrease in viable BMSCs. This indicates that the lowest CuCl_2_ dose used (Group C) positively affected BMSC proliferation, whereas the other two doses (Groups D, E) impaired it. When seeded after pre-elution in DMEM for 6 h (i.e., Scenario 2), all groups supported BMSC proliferation between days 1 and 5 (Figure 5b), with peak values from Group C. At all time points, Group E became moderately higher than Group A, suggesting a substantial loss of cytotoxicity to BMSCs.

Under Scenario 1, the expression levels (Figure 5c) of the four osteogenic genes varied in n-shaped profiles, with peaks observed from Group C. The expression levels of Group E returned to below those of Group A. Under Scenario 2, the expression levels of the osteogenic genes (Figure 5d) also varied in n-shaped profiles peaking at Group C. However, in this scenario, the decrease in the expression level from Group C to E became more moderate, and the levels of Group E were significantly higher than those from Group A (all *p* < 0.05).

### 3.4. In Vivo Anti-Infection and Fracture Healing

**Figure 6:**
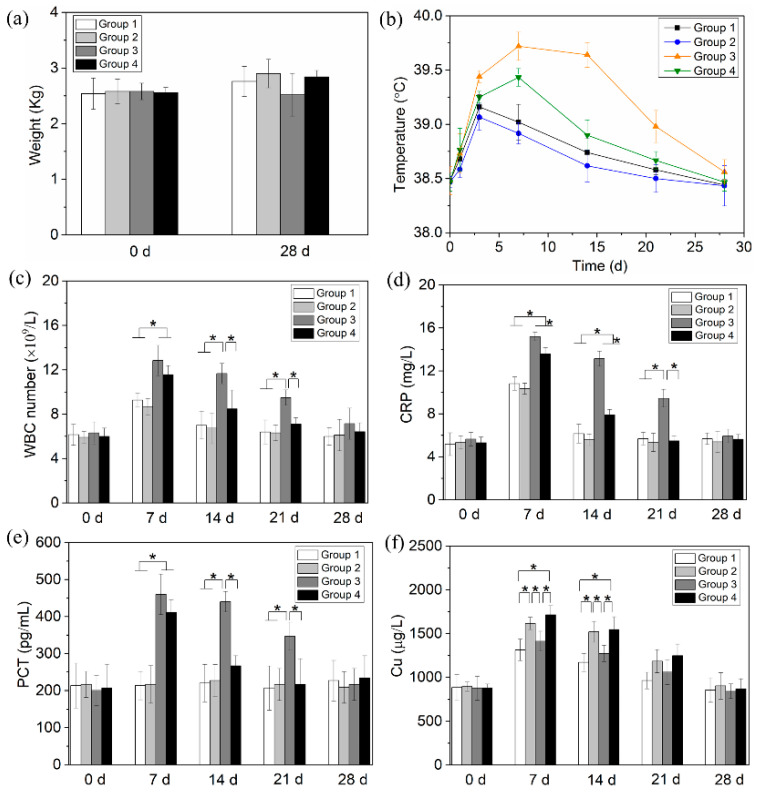
(**a**) The weight, (**b**) temperature, and (**c**–**f**) blood analysis of animals in various groups (* represents *p* < 0.05).

Before operation, there was no significant difference among the groups in body weight (*p* = 0.99, Figure 6a); on day 28 (i.e., end of study), Group 3 was moderately lighter than the other three groups but the difference was not statistically significant (*p* = 0.18). One animal in Group 3 developed diarrhea and died on day 14. All other animals survived during the study.

The rectal temperatures of all groups varied in an n-shaped profile (Figure 6b); Groups 3 and 4 reached peak temperatures on day 7, with significantly higher values than the other two groups. The peak temperature of Group 3 was also significantly higher than that of Group 4 (*p* = 0.001). WBCs of all groups also followed an n-shaped profile (Figure 6c), peaking on day 7. Compared with Groups 1 and 2, Group 3 had significantly higher WBCs between days 7 and 21, and Group 4 had significantly higher values on days 7. Between days 7 and 21, Group 3 was also significantly higher than Group 4. CRP (Figure 6d) varied in a similar pattern to the evolution of WBC. PCT of Group 3 (Figure 6e) was significantly elevated (vs. Groups 1 and 2) between days 7 and 21, and Group 4 did so on day 7. Between days 7 and 21, Group 3 was also significantly higher than Group 4. Blood copper levels of all groups (Figure 6f) also varied in an n-shaped profile, peaking on day 7. Between days 7 and 14, Groups 2 and 4 (i.e., CuCl_2_-loaded groups) were significantly higher than the other two groups. On day 28, all these indicators were renormalized (vs. day 0).

**Figure 7:**
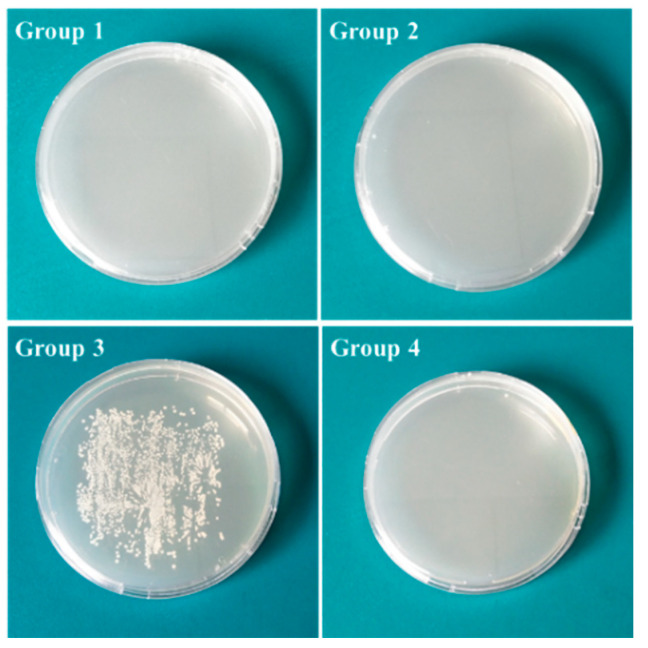
Roll-over cultures of different Ti implants after 24 h of incubation.

After incubation, the agar inoculated with smear prepared from wires of Group 3 formed white colonies (Figure 7). No wires from other groups formed visible colonies on agar medium.

**Figure 8:**
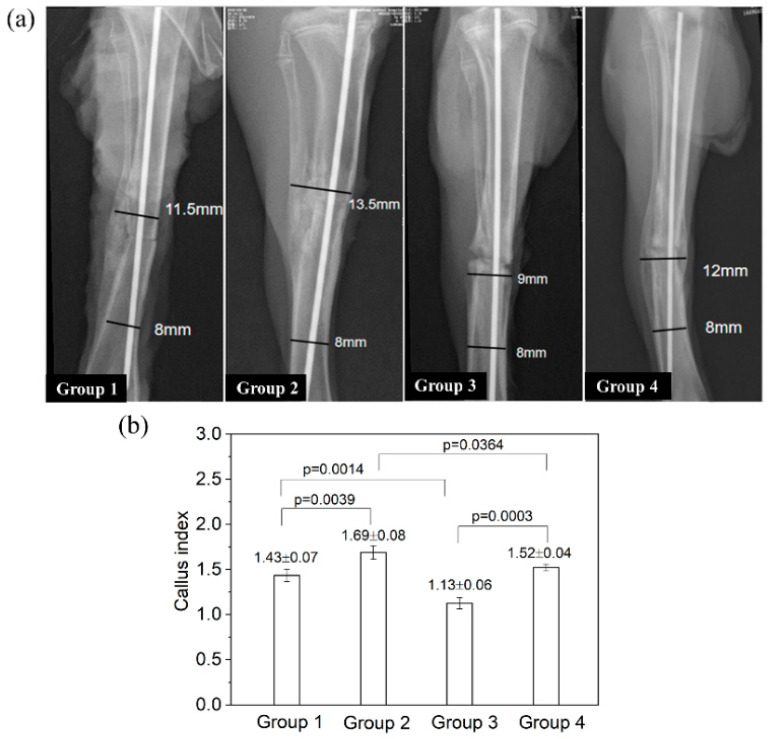
The radiographic examinations of rat tibia after implant removal on day 28. (**a**) The representative images of the callus formation in different groups. Lines and numbers indicate how the callus index was determined. (**b**) The value of callus index for different groups.

On postmortem radiographs (Figure 8a), Group 1 (blank control) showed a clear periosteal reaction (see the left side of the tibia) and extensive callus formation bridging the fracture ends. Group 2 had a weakly visible fracture line and satisfactory cortex continuity, suggesting a faster union (vs. Group 1). In comparison, Group 3 displayed a wide fracture gap with signs of bone resorption at the fracture ends, showing a delayed union. Group 4 had a narrower fracture line (vs. Group 3) and extensive callus bridging the gap, indicating a status of healing superior to Group C but inferior to Group B. The callus index of Group 3 was significantly lower than that of all other groups (all *p* < 0.01; Figure 8b). The callus index of Group 2 was significantly higher than that of Group 1 (*p* = 0.0039); the callus index of Group 4 was slightly higher than that of Group 1, but the difference was not statistically significant (*p* = 0.377).

**Figure 9:**
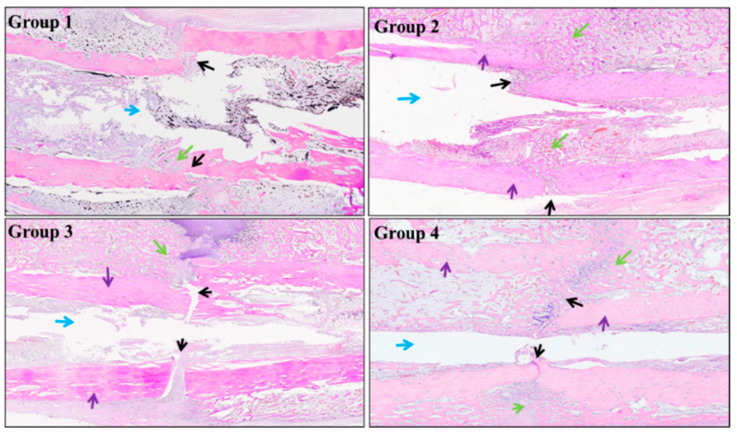
Representative histological images after implant removal on day 28 (black arrows: bone fracture line; green arrows: new bone; blue arrows: bone marrow cavity; purple arrows: cortical bone).

Histological examinations (Figure 9) revealed that, in Group 1, the fracture ends were connected by callus containing small amounts of trabecular bone and chondrocytes. In Group 2, extensive callus bridged the gap, with massive trabecular connection. Intramembranous and extra-membranous osteogenesis were visible, with abundant neo-vessels in the new bone tissue, indicating active bone remodeling. In Group 3, the fracture gap was evident and infiltrated by fibrous tissues; no clear intra-membranous osteogenesis was observed at both sides of the fracture ends; only extra-membranous osteogenesis was seen at one side of the fracture end, indicating an early stage of bone remodeling. In Group 4, the fracture gap was filled by large amounts of callus and some cartilaginous tissue; neo-vessels occurred in the new bone tissue, demonstrating ongoing remodeling. Accordingly, the fracture repair score was 3 for Group 1, 3–4 for Group 2, 0–1 for Group 3, and 3 for Group 4, respectively.

## 4. Discussion

In this study, Cu^2+^ release from the simple CuCl_2_-loaded PLA coating produced antibacterial and pro-osteogenic effects in vitro and in vivo. Earlier studies have identified multiple mechanisms underlying the antibacterial actions of Cu^2+^ [20], and different bacterial species are likely killed predominantly by different mechanisms. For *E. coli*, Cu^2+^ was reported to induce the oxidation of unsaturated fatty acids in the cell membrane, leading to membrane damage and bacterial death [20,21]. In comparison, Cu^2+^ was believed to kill *S. aureus* primarily by triggering DNA breakdown, without causing membrane disintegration [20]. Nevertheless, other bactericidal mechanisms have also been identified, and their relative contribution requires further investigation.

The mechanisms responsible for the pro-osteogenic effect of Cu^2+^ are not understood but, as suggested by Rodrigues et al. [22], may involve increased maturation of the extracellular matrix. They reported that Cu^2+^ (5 or 50 µM Cu–histidine complex) stimulated the osteoblastic differentiation of BMSCs isolated from postmenopausal women but reduced their proliferation and ALP expression. In comparison, we observed increased osteoblastic differentiation and viability of rabbit BMSCs exposed to Cu^2+^-release coatings. The different trends in viability/proliferation between the studies are unclear but may, at least, include differences in experimental conditions, such as cell characteristics and the concentration and chemistry of Cu compounds (i.e., CuCl_2_ vs. Cu–histidine).

A number of metallic elements have been reported to have antibacterial activities, as recently reviewed [23]. Of these, Ag and Cu are among the most intensively studied. When Ag^+^ and Cu^2+^ were compared, the minimally inhibitory concentrations (MICs) of Ag^+^ were approximately two orders of magnitude lower than Cu^2+^, for both *E. coli* and *S. aureus* [23], indicating the former to be a more potent ion. In comparison, Kawakami et al. evaluated the antibacterial activities of 21 pure metals [24], and observed Ag and Cu to be similarly effective to both *E. coli* and *S. aureus*, with minor differences. The difference between ions and metals is explained by the higher corrosion resistance (i.e., ion dissolution) of solid Ag (vs. Cu). Additionally, Cu is an essential element for humans and thus features a relatively higher degree of safety (LD50 for the rat: 359.6 mg/kg) compared with Ag. To our knowledge, no Cu-coated orthopedic implants have been commercialized; however, copper intrauterine devices have been widely used for contraception for decades, with satisfactory safety records [25], suggesting the potentially safe use of Cu for implantable devices.

In the present study, Group E contained ~3 mg of Cu^2+^ and ~88% of this was released in vitro within 24 h. In vitro, Group E released ~91% of the CuCl_2_ loaded within 48 h, followed by a negligible subsequent release (Figure 2). This immediate burst release is expected to be desirable for the prevention of IPI [26]. The CuCl_2_ particles used in our study were several micrometers in size. Particle size distribution is likely a factor influencing the release kinetics of Cu^2+^, but this was not investigated. More studies are needed to characterize the release kinetics of a range of particle sizes (e.g., nano- to micrometers) and determine the optimal ones. Additionally, the CuCl_2_ particles were relatively homogeneously embedded in the coating, without severe agglomeration (Figure 1). This may be partly due to the sonication of the coating solution before dip-coating. A homogeneous particle distribution is important for a reproducible release kinetics, as agglomeration changes the surface available for ion dissolution and subsequent diffusion in the liquid.

The coatings killed 68.6–96% of the *S. aureus* after 2 h in liquid medium depending on the CuCl_2_ loaded, and killed 95.4–99.7% after 6 h. The inhibition rates reported in the literature varied substantially, partly because different studies used different experimental protocols (e.g., bacterial strain, conditions of exposure to Cu, time points studied, method of quantitation). This makes a direct comparison of results between studies difficult. For example, Wan et al. reported inhibition rates of 31–100% from Cu^2+^-implanted Ti after 24 h exposure to *S. aureus*, depending on the dose implanted [12]. Wang et al. recorded inhibition rates of 41.5–99.4% from Ti modified by a polydopamine coating and Cu^2+^ adsorption after 24 h, depending on the amount of Cu^2+^ adsorbed [14]. Interestingly, Hoene et al. studied the antibacterial activities of Cu-coated Ti between 2 and 24 h [27]. They galvanically deposited metallic Cu on Ti, and grit-blasted the surface to leave 1 µg·mm^−2^ Cu. The surface reduced the *S. aureus* number by ~1 log at 2 h, and ~2 logs at 6 h; these results are relatively consistent with our findings. Clinically, the first 6 h after the implantation operation was suggested to be a “decisive period” for the occurrence of implant-related infections, because the introduced pathogens are quiescent during this period, making it a critical opportunity to kill pathogens near the implant [28,29]. Therefore, the effective bacterial inhibition within 6 h found in the present study (Figure 4) may be clinically meaningful.

In the in vivo model, all groups had elevated serum Cu levels (vs. day 0) that peaked on day 7 and renormalized between days 21 and 28 (Figure 6). Because this transient elevation was also seen even in Group A, which received no Cu-release coating, this can only be explained by the increased absorption of Cu from the diet. This suggests an increased requirement of Cu by the animals for bone healing, as also reported by others [26]. Furthermore, between days 7 and 21, Groups 2 and 4 showed higher serum Cu levels than Groups 1 and 3, corresponding to the Cu^2+^ released from the coating. In all groups, the serum Cu level peaked on day 7; however, the in vivo release profile could not be estimated because of the paucity of data between days 1 and 7. Nast et al. [26] evaluated the release of gentamicin from a PLA coating on Ti K-wires placed in the medullary cavities of rats, and found a high concentration of gentamicin in the adjacent endosteum 1 h after implantation, indicating fast in vivo release, similar to that recorded in vitro [4]. Direct data on in vivo Cu release are unavailable from the present study; however, considering the similarity between the physical nature of the reported gentamicin–PLA coating [4] and our CuCl_2_–PLA coating (i.e., soluble particles dispersed in a PLA film and released by dissolution via microscopic pores/defects in the film), a burst release similar to the in vitro pattern (Figure 2) can be expected. Nevertheless, to elucidate the rate of in vivo Cu^2+^ release, more measurements will be performed within day 1.

The transient elevation of serum Cu resulting from the Cu-release coating is inevitable, and its impact is unknown. The normal range of serum Cu in adults is ~750–1450 µg/L. In pregnant women, plasma copper was reported to be approximately one-fold higher than in unpregnant individuals [30]. In our study, on day 7 (peak point of serum Cu), Group 1 had ~49% higher serum Cu compared with day 0, and Groups 2 and 4 had ~23% and ~31% higher values than Group 1. Considering these, the transient elevation may be considered safely tolerated by the body. Nevertheless, one advantage of the simple coating design is the relative ease of adjusting the dose of CuCl_2_ loaded. This may be achieved by controlling the CuCl_2_ concentration in the PLA solution and the dip–withdraw speed.

PLA is a hydrophobic polymer widely reported to generate reduced cell adhesion. Thus, the significantly increased viability of BMSCs inoculated on the blank PLA coating relative to those on Ti discs (Figure 5, Group B vs. Group A) seemed surprising. The mechanisms are not fully understood, but a tentative explanation may be suggested. As all discs were disinfected by UV irradiation in the air for 24 h before cell inoculation, the surface of the PLA coating was partly oxidized into hydrophilic groups (e.g., hydroxyl, carboxyl); as a result, the coating partly lost its hydrophobicity. Additionally, Wurn et al. also reported the significantly higher proliferation of human osteoblasts cultured on PLA discs prepared by fused deposition (vs. on Ti control) between days 1 and 10 [31]. However, considering the progressive hydrolytic degradation of PLA in vivo and the formation of a fibrous capsule [32], the coating may not provide the stable, strong osseointegration required by permanent implants (e.g., artificial joints). Therefore, the coatings reported here are expected to be suitable for osteosynthetic devices, such as intramedullary nails and fixation plates.

Although positive results have been obtained from the present study, more critical insights are needed before the coating can be considered for actual applications. The potential effects of the coating on major organs (e.g., liver, kidney) need to be clarified. Furthermore, the minimal dose that generates a clinically significant antibacterial effect without introducing observable toxicity should be established. These will be considered in future studies.

## 5. Conclusions

PLA coatings containing three concentrations of Cu^2+^ were prepared on Ti by dip coating in solutions containing CuCl_2_ particles. All coatings produced rapid Cu^2+^ release during the first 6 h. When BMSCs were directly seeded on the coatings, the cell viability decreased Cu^2+^-dose-dependently compared with the Cu^2+^-free coating. When the cells were seeded after pre-elution in DMEM for 6 h, the dose-dependent pattern remained. However, in this scenario, the cell viability on the coating containing the lowest Cu^2+^ concentration became significantly higher than those on uncoated Ti and the Cu^2+^-free coating; the viability on that containing the highest Cu^2+^ concentration became similar to the uncoated Ti, suggesting the loss of cytotoxicity. Agar diffusion and plate counting tests confirmed all coatings to be effective at inhibiting *S. aureus* growth. The coating containing the highest Cu^2+^ concentration was prepared by K-wires and tested in a tibial internal fixation model challenged with *S. aureus*. The coating eradicated *S. aureus* infection and accelerated fracture-end union and callus formation. These results suggest the coating to be a feasible technique for reducing postoperative infection associated with orthopedic implants.

## Figures and Tables

**Table 1:** Sequences of primers for bone-related genes.

Gene	Forward Primer (5′-3′)	Reverse Primer (5′-3′)
ALP	GTTCAGTGCGGTTCCAGACAA	CTGCAAGGACATCGCTTATCA
OPN	TGGCTTTCAATGGACTTACTCG	CAGCTTTACCACAAACACCCG
OCN	TGATGCAAGCCTGACCTCC	CCATAGCCCACGGCCAAAA
OSX	GCAAGGTGTACGGCAAGG	CAGAGCAGGCAGGTGAATT

**Table 2:** Groups for in vivo anti-infective model.

	PDLLA Coating (CuCl_2_-Free) †	PDLLA Coating (1.0 mg/mL CuCl_2_) ‡
Inoculating normal saline	Group 1	Group 2
Inoculating *S. aureus* (10^5^ CFU/mL × 100 μL)	Group 3	Group 4

† Prepared following Section 2.1, Group B. ‡ Prepared following Section 2.1, Group E.

## Supplementary Materials

The following supporting information can be downloaded at: https://www.mdpi.com/article/10.3390/jfb13020078/s1, Figure S1: (a) Water contact angles and (b) surface roughness (Ra) of different samples; Figure S2: SEM micrographs of samples from Groups A and E after immersion in DMEM for 2 d; note 1) the smoother and more featureless surface (i.e., coating) on Group E sample vs. the grainy texture of Group A; 2) two circular structures on Group E sample are the “footprints” of two original CuCl2 particles dissolved during immersion; Figure S3: SEM images of Ti discs after co-culture with *S. aureus* for 6 h.
