# Supplementary material for: Cu2+ Release from Polylactic Acid Coating on Titanium Reduces Bone Implant-Related Infection
Source: J Funct Biomater. 2022 Jun 10;13(2):78. doi: 10.3390/jfb13020078 (PMC9225639; doi:10.3390/jfb13020078)

Supplementary Figure S1 (a) Water contact angles and (b) surface roughness (Ra) of different samples.

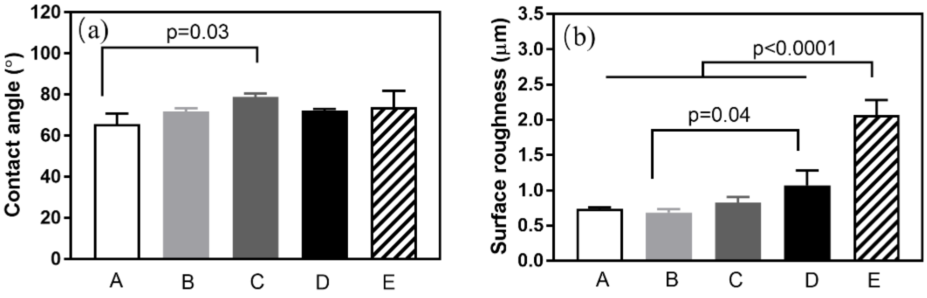

Supplementary Figure S2 SEM micrographs of samples from Groups A and E after immersion in DMEM for 2 d; note 1) the smoother and more featureless surface (i.e., coating) on Group E sample vs. the grainy texture of Group A; 2) two circular structures on Group E sample are the "footprints" of two original $CuCl_2$ particles dissolved during immersion.

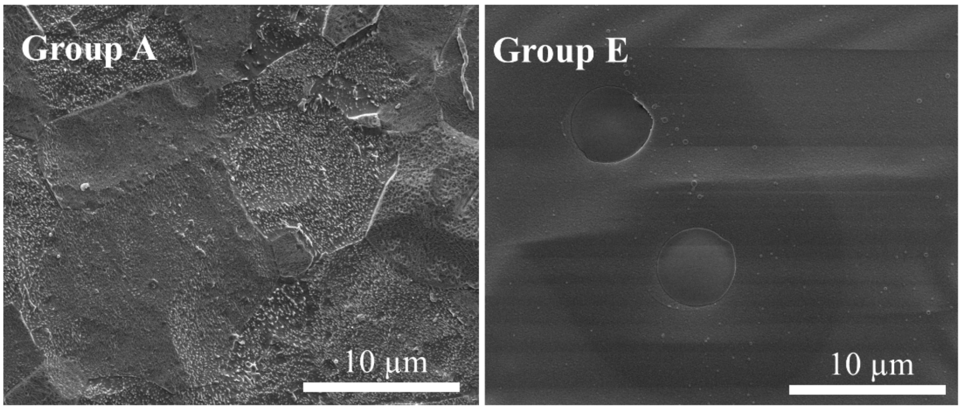

Supplementary Figure S3 SEM images of Ti discs after co-culture with *S. aureus* for 6 h

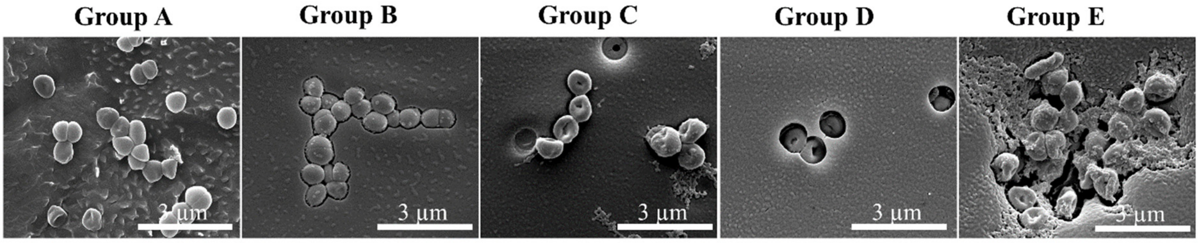

Supplement: Supplementary file 1 [file jfb-13-00078-s001.zip › jfb-1735355-supplementary.pdf]
